# Leisure in Children and Adolescents with Juvenile Idiopathic Arthritis: A Systematic Review

**DOI:** 10.1371/journal.pone.0104642

**Published:** 2014-10-20

**Authors:** Sabrina Cavallo, Karine Toupin April, Viviane Grandpierre, Annette Majnemer, Debbie Ehrmann Feldman

**Affiliations:** 1 Département de Médecine Sociale et Préventive, École de Santé Publique, Université de Montréal, Montréal, Québec, Canada; 2 Montreal Children's Hospital, McGill University Health Center, Montréal, Québec, Canada; 3 Institut de Recherche en Santé Publique Université de Montréal, Montréal, Québec, Canada; 4 Centre de Recherche Interdisciplinaire en Réadaptation du Montréal Métropolitain, Institut de Réadaptation de Montréal, Montréal, Québec, Canada; 5 Children's Hospital of Eastern Ontario Research Institute, Ottawa, Ontario, Canada; 6 Department of Pediatrics, Faculty of Medicine, University of Ottawa, Ottawa, Ontario, Canada; 7 School of Rehabilitation Sciences, Faculty of Health Sciences, University of Ottawa, Ottawa, Ontario, Canada; 8 School of Physical and Occupational Therapy, Faculty of Medicine, McGill University, Montréal, Québec, Canada; 9 École de Réadaptation, Faculté de Médecine, Université de Montréal, Montréal, Québec, Canada; 10 Direction de Santé Publique de Montréal, Montréal, Québec, Canada; Faculté de médecine de Nantes, France

## Abstract

The aim of this systematic review is to describe participation in social and physical leisure activities among children and adolescents with JIA, as well as identify potential determinants of leisure participation.

**Methods:**

Electronic databases were systematically searched for articles published up until June 2013 pertaining to participation in leisure activities among youth with JIA and other rheumatic diseases. Studies were included if they measured involvement in either social or physical leisure activities. Selection and quality appraisal of articles were completed independently by two authors.

**Results:**

Eight hundred and ninety-three articles were found through electronic and reference search. One hundred and nine full articles were reviewed to assess for eligibility. Twelve articles met inclusion criteria and findings were reviewed. Most focused on describing participation in physical rather than social activities. Results suggest that youth with JIA participated less in both social and physical leisure activities as compared to healthy peers, and those with JIA did not meet national recommendations for physical activity. Potential determinants of leisure participation were socio-demographic (age, sex), anthropometric (height, weight) and disease-related (JIA subtype, disease duration, pain, number of swollen or painful joints, stiffness, fatigue, well-being) factors.

**Conclusion:**

Characterization of leisure activity remains limited and mostly focused on physical activity in JIA. Assessment of more comprehensive outcome measures is warranted to obtain a better description of leisure in this population. Evidence of the influence of contextual factors as potential determinants of involvement in leisure among children with pediatric rheumatologic diseases is needed.

## Introduction

Juvenile idiopathic arthritis (JIA), which includes juvenile rheumatoid arthritis (JRA), is one of the most common chronic conditions of childhood [1]. Children and adolescents with JIA are at greater risk for adopting a more sedentary lifestyle compared to their healthy peers in part due to disease related factors such as pain, fatigue, swollen and stiff joints [1], [2]. In the past decade there has been a growing interest for the study of leisure participation in children with disabilities. However this concept has been vastly understudied in youth with pediatric rheumatologic diseases.

Leisure participation has been defined as the ‘involvement in the formal and informal everyday activities of childhood in all types of non-school environments, including environments for play, sport, entertainment, learning, and religious expression’(King et al., 2003, p. 65) [3]. Participation in leisure activities is of critical importance in childhood and adolescence to maintain a fit lifestyle, develop friendships, engage in focus-oriented activities, as well as acquire cognitive and social skills important for development [4]–[6]. If participation in leisure activities remains limited on a long-term basis, children and adolescents may not have enough social contacts with peers, may be less able to make friends, experience greater social isolation, and may be at greater risk for depression [7]. Furthermore, engagement in active physical activities is important for physical and cardiovascular health.

Participation is a key component of the International Classification of Functioning, Disability and Health (ICF) endorsed by the World Health Organization (WHO) [8]. As depicted in the ICF, a child's participation in leisure may be influenced by various factors to include those related to the health condition (e.g. disease severity), as well as personal (e.g. age, sex) and environmental (e.g. accessibility to services) [8], [9].

Most of the existing literature in JIA focuses on describing the level of involvement and benefits of exercise or physical activity programs on health outcomes such as quality of life, physical function and fitness, as well as other JIA related disease outcomes (e.g. number of swollen joints and bone mineral density) [10]–[14], as well as describing the level of involvement. However limited research has been done in the broader area of leisure participation in JIA.

The main purpose of this systematic review was to describe involvement in social (e.g. outings with friends and/or family) and physical (e.g. sports, biking, swimming, etc.) leisure activities among children and adolescents with JIA. It reports on the type and frequency of these activities as compared with either healthy controls, normative data or health guidelines. A secondary objective was to identify potential socio-demographic, disease-related, personal and environmental determinants of leisure activities in children and adolescents with JIA.

## Materials and Methods

### Literature Search

The first author (SC) devised an electronic search strategy (Appendix S1) in collaboration with a librarian. The librarian also assisted in identifying the appropriate key terms for the systematic review and reviewed the final search strategy to ensure correctness. An example of the detailed search strategy formatted for the MEDLINE database with all key words is provided (Appendix S1). In addition to MEDLINE (1946 to Present), we searched the following electronic databases: CINAHL (1982 to December week 1 2013), Base de Données en Santé Publique (June 2013), ERIC (1965 to April 2013), Health and Psychosocial Instruments (1985 to April 2013), OT Seeker (June 2013), PsycINFO (1806 to May Week 3 2013), EMBASE (1974 to 2013 Week 21), Cochrane Database of Systematic Reviews (2005 to March 2013), ACP Journal Club (1991 to April 2013), Database of Abstracts of Reviews of Effects (2nd Quarter 2013), Cochrane Central Register of Controlled Trials (March 2013) and Cochrane Methodology Register (3rd Quarter 2012). Key term selection was guided by using a PICOS (populations, interventions, comparators, outcomes, study design) framework [15]–[17]. The search strategy did not restrict on language or design of the study; non pertinent articles were sifted out by authors after the search was completed. Scientific journals, internet browsers and reference lists of reviewed articles were also consulted for any pertinent information and potential articles.

### Inclusion and Exclusion Criteria

We included studies related to participation in physical or social leisure-time activities completed by children between the ages of 0 and 21 years diagnosed with juvenile idiopathic arthritis, juvenile chronic arthritis, or juvenile rheumatoid arthritis. All diagnostic classifications provided by the International League of Associations for Rheumatology (ILAR), the European League Against Rheumatism (EULAR) or the American College of Rheumatism (ACR) were accepted. As both social and physical activities are considered leisure pursuits we have focused on including a variety of habitual activities performed for the purpose of having fun. Studies reporting findings exclusively on participation in exercise programs done during school classes or as part of a regimented exercise program in a controlled setting (laboratory) were excluded. Both English and French publications were included in the search. We restricted our search to quantitative studies. Although reviewed for pertinent information and potential references, review papers, abstracts, commentaries or letters to the editor, study protocols, work group or conference proceedings and studies aimed at validating measures were not retained for the systematic review. Results from our search were exported into EndNote X6 and subsequently managed in excel database sheets.

### Identification of Studies

After duplicates were removed, two authors from the team (SC and VG) independently screened titles, abstracts and key words for pertinent articles according to the identified eligibility criteria. After initial screening the retained full articles were assessed to ensure that they met inclusion criteria and a list of relevant articles was compiled. A review of these articles was completed by each reviewer who then independently decided which articles met the eligibility criteria and should be included in the systematic review. If consensus could not be reached between reviewers, a third author (KTA) resolved any disagreements.

### Data Extraction

Two authors (SC and VG) independently extracted data from the retained articles using a previously pilot-tested extraction table. After thorough review of selected articles information on the studies (study design, geographical location, sample size, participant characteristics [i.e. age and sex distribution, diagnosis], objectives) and the methods of data collection (measure of leisure participation, child or proxy report, psychometric properties) were summarized in a table, as well as information on potential determinants of leisure participation was reported in a separate table. The Preferred Reporting Items for Systematic Reviews and Meta-Analyses (PRISMA) statement was used to inform reporting of this systematic review (see completed PRISMA checklist in Appendix S2) [18].

### Study appraisal

Studies meeting the inclusion criteria were systematically appraised using the Quality Assessment Tool for Quantitative Studies [19]. This tool has been deemed suitable for quality assessment of randomised and non-randomised studies [20]. Ratings of strong, moderate or weak were assigned for each of the following six quality components: selection bias, study design, confounders, blinding, data collection methods, withdrawals and dropouts [21]. The tool's test-retest (intra-rater) reliability was assessed for two reviewers and results on agreement were acceptable (Kappa 0.74 and 0.61, respectively for each reviewer) [19]. Adequate content and construct validity were also demonstrated [19].

### Synthesis of findings

A narrative summary allowed us to analyse our findings by describing content and highlighting strengths and weaknesses of reviewed studies. Due to the heterogeneous characteristics of the included studies (demographic distributions, leisure outcomes, units of measure) it was not feasible to conduct a meta-analysis [22].

## Results

**Figure 1:**
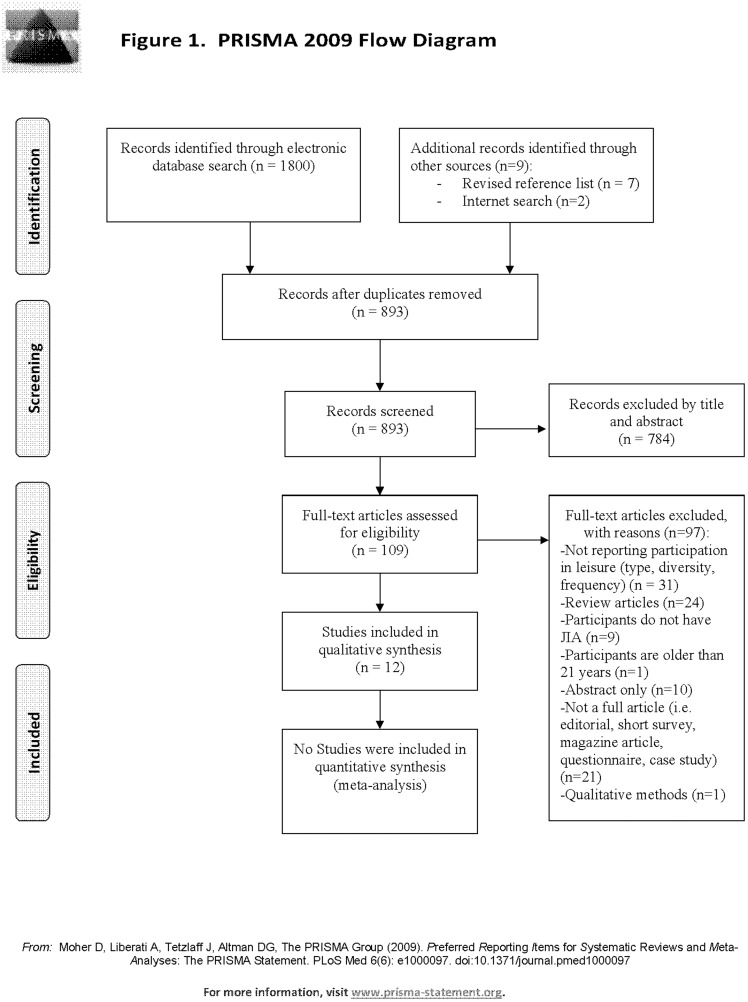
PRISMA Flow Diagram of Included Articles. Figure 1 presents a PRISMA flow diagram of articles included in the systematic review as well as the main reasons for rejection.

The electronic search yielded 884 unique references (i.e. after duplicates were removed). An additional 9 references were obtained through manual review of reference lists from consulted articles and through internet browser search. A total of 893 titles and abstracts were screened. One hundred and nine full articles were reviewed to assess for eligibility. Of these, 12 articles met inclusion criteria and were included in the qualitative synthesis of results (Figure 1).

The identified studies were rated as to their quality. Using the Quality Assessment Tool for Quantitative Studies, none of the studies met all of the quality criteria [19], [21]. All studies exhibited some level of selection bias. Selected participants were deemed to be at most somewhat likely to represent the studied population, appraisal scores ranged from weak (n = 2) [23], [24] to moderate (n = 10) [2], [25]–[33]. Retained studies were observational. Half of the studies adequately controlled for important confounders [25], [26], [30]–[33], such as age and sex. Only 3 studies collected data on leisure-time activities using outcome measures with known or properly cited validity and reliability [23], [28], [30]. The descriptive characteristics of study participants and information on leisure participation are included in Table 1. Studies reporting on potential determinants of leisure participation are described in Table 2.

**Table 1:** Findings of systematic review on leisure participation in children and adolescents living with juvenile arthritis.

Author, Country (year)	Participants		Study design	Leisure outcome measure	Variable recorded	Findings
	JIA	Comparator groups				
Billings, USA (1987)	56 (37 girls and 19 boys); mean age 13.7 years (≥10 years)	Within group comparison and 77 HC	Cross-sectional	Youth Health and Daily living form	# of social activities done with family, friends and at school out of 10	Fewer family activities for severe JIA group compared to those with mild/inactive JIA (p<0.05). Fewer activities with friends for JIA groups compared to HC (p<0.05). More family activities for mild/inactive JIA compared to HC (p<0.05).
Félin, USA (2007)	48, age range 4–18 years	Within group comparison and 266 HC	Cross-sectional	PA questionnaire on weight-bearing activities	Average METs hr/wk in the last year	Decreased weight bearing PA for systematic JIA group compared to oligoarthritis, polyarthritis JIA or HC groups (p≤0.01). No significant differences between oligoarthritis or polyarthritis JIA groups and HC.
Henderson, USA (1995)	23 (16 females), mean age 8.1 years (range 5–11 years	23 HC	Cross-sectional	Caltrac accelerometer and UCMS; Parent-report activity record, 3 day; Parent-report questionnaire on organized sports	Average daily movement counts; Daily activities, type (light, strenuous) and intensity (hours/day); Frequency (hours/week) and amount in the last year (months)	No significant differences in daily movement. Decreased time spent in strenuous activities for JIA group (p<0.01). No significant differences in low intensity PA between groups. ↓ time spent participating in sports at baseline (p = 0.01) and in the last year (p<0.01) for JIA group.
Henderson, USA (1997)	48 (37 female, 11 male), mean age 8.1±1.9 years (range = 4.6–11.0 years)	Within-group comparisons	Cross-sectional	Caltrac accelerometer and UCMS; Parent-report activity record, 3 day; Parent-report questionnaire on organized sports	Average daily movement counts; Daily PA, type (light, strenuous) and intensity (hours/day); Frequency (hours/week) and amount in the last year (months)	No significant differences in daily movement. No significant differences in daily PA. Decreased time spent in organized sports for JIA group with low TB BMD compared to those with normal TB BMD (p = 0.03).
Henderson, USA (2000)	Thirty-six females, mean age 16.0±1.8 years (age range 11 to 18 years)	Within-group comparisons and 51 HC	Cross-sectional	Caltrac accelerometer and UCMS; Self-report activity record, 3 day; Self-report questionnaire on organized sports	Average daily movement counts; Daily PA, type (light, strenuous) and intensity (hours/day); Frequency (hours/week) and amount in the last year (months)	No significant differences in daily movement. No significant differences in daily PA. 50% of JIA patients participated in organized sports compared to 65% of HC (p = 0.01). Decreased time spent in organized sports for JIA group compared to HC (p = 0.005).
Huygen, Netherlands (2000)	47 (32 girls and 15 boys), age range 7–16 years (child, 7–11 years of age; adolescents, 12–16 years of age)	52 HC	Cross-sectional	Dutch CBCL, parent-report on the child's social functioning; Dutch Youth Self Report, adolescents self-report on social functioning	3-point Likert scale	Decreased participation in play with friends for children with JIA (p = 0.04). No significant differences between groups for seeing friends. Decreased participation in sports for adolescents with JIA (p = 0.00).
Lelieveld, Netherlands (2008)	30 (18 girls, 12 boys), mean age 17.0±0.6 years	106 HC	Cross-sectional	Self-report activity diary, 3 days	Daily average PA (hours/day)	Decreased PA in JIA group (p<0.01). Increased time spent in bed for JIA group. No significant differences in PA between boys and girls among JIA or HC groups. No significant differences in low intensity activities between JIA and HC. Decreased time spent in moderate intensity leisure activities (p<0.01); high intensity leisure activities (p<0.05); and in competitive sports (p<0.01) for JIA group. Only 23% of patients with JIA met public health recommendations to perform ≥1 hour daily MVPA (mean of 87 min/day of MVPA) compared with 66% of HC (mean of 133 min/day of MVPA).
Lien, Norway (2005)	108, age range of 6–18 years	108 HC	Longitudinal	Self-report questionnaire on weight-bearing activities outside of school hours	Frequency (number of times per week), ordinal scale (0.5 = less than once a week, 1 = once a week)	Decreased participation in weight-bearing activities at baseline (p = 0.033) and at the 2 year follow-up (p = 0.040).
Maggio, Switzerland (2010)	31, mean age 10.8±0.5 years (range 4.8 to 17.9 years)	85 HC, 45 obese, 48 type 1 diabetes mellitus	Cross-sectional	Uniaxial Actigraph accelerometer, 7 days	Average daily PA (minutes/day)	When adjusted for age, decreased daily PA for JIA group compared to HC (p<0.001) and compared to patients with obesity (p = 0.002). Decreased time spent in MVPA for JIA group versus HC (p = 0.036). 38.1% of JIA, 38.5% of type 1 diabetes mellitus, 51.6% with obesity and 60.4% of HC met the daily recommended 60 minutes of MVPA.
Schanberg, USA (2003)	41 (59% were girls), mean age 12.3±2.9 years (range 8–17 years)	None	Longitudinal	Self-report diary on daily social activities	Reduction in activities, 4-point Likert scale (anchored by ‘not at all’ and ‘a lot’)	No control data available for comparison
Takken, Netherlands (2003)	45 (10 male, 35 female), mean age 8.9±2.2 years	None	Cross-sectional	Caltrac activity monitor measuring daily PA for 4 consecutive days; Parent-report PAL	Average daily motion counts; Child's usual PAL, 5 point Likert scale (1 = inactive to 5 = very active)	No control data available for comparison
Tarakci, Turkey (2011)	52 (33girls, 19 boys), mean age 12.13±2.92 years (range 8–17 years)	48 HC	Cross-sectional	Self-report diary on PAL, 1-day	METs/day	↓ time spent in PA for JIA group (p = 0.000)

JIA, Juvenile idiopathic arthritis; UCMS, University of Cincinnati Motion Sensor; HC, Healthy controls; PA, Physical activity; PAL, Physical activity level; TB BMD, Total body bone mineral density; CBCL, Child Behaviour Checklist.

**Table 2:** Potential determinants of participation in leisure-time activities in JIA identified in the systematic review.

	Potential determinants	Association with leisure
Socio-demographic		
	Age [29]	Older age was associated with ↓ PA
	Sex [28]	Male sex was associated with ↑ PA level
Anthropometric		
	Weight [28]	↑ weight was associated with ↑ PA
	Height [28]	↑ height was associated with ↑ PA
Disease		
	Disease duration [32]	Longer disease duration was associated with ↑ PA
	Pain [2]	↑ perceived pain and the number of painful locations were associated with ↓ social activity
	Stiffness [2]	↑ daily stiffness was associated with ↓ social activity
	Fatigue [2]	↑ fatigue was associated with ↓ social activity
	Swollen joints [29]	↑ number of swollen joints was associated with ↓ PA
	Physical fitness** [29]	↑ maximal oxygen consumption (absolute, relative) was associated with ↑ PA
	Well-being [32]	↓ perceived well-being was associated with ↓ PA

PA, Physical activity.

**The association between physical fitness and physical activity may be bidirectional, i.e. physical fitness can be both a determinant and an outcome of PA [29].

### Characteristics of the Studies

Only one study included data from a childhood rheumatic disease database [23], while all others recruited participants from tertiary care centers (hospital and centers). One study included 78 participants with JIA and 17 with other rheumatologic diagnoses [25], whereas all other studies included only participants with JIA. A range of ages were studied (4 to 18 years); certain authors focused their examination of leisure participation on specific age groups: children (n = 3) [24], [27], [30] and/or adolescents (n = 3) [25], [26], [30], whereas other studies reported outcomes for all ages combined (e.g. 4 to 18 years) (n = 5) [2], [23], [28], [31], [33]. Ten out of the 12 studies used comparison analysis to illustrate differences in leisure participation between groups (within JIA group analysis (n = 4), healthy controls (n = 9) and controls with other medical conditions (n = 1)). Of these, only one study employed matching on age and sex, as well as race and county of residence [31], while others either controlled for covariates such as age, sex and anthropometric factors (weight, height or body mass index) [23], [24], [26], [28], [32], [33], and others simply reported findings in groups of similar age and sex [25], [30].

The main objective of 8 studies hinged on reporting information on the level of participation in leisure activities among those living with JIA, as well as identifying potential determinants and comparing findings on leisure participation between patients with JIA and controls [2], [24], [25], [28]–[30], [32], [33]. The remaining 4 studies inquired primarily on overall bone health (geometry, density and strength) and participation in leisure-time physical activity as a secondary outcome [23], [26], [27], [31].

Participation in social activities was assessed through self-report and quantified using various scales (e.g. Likert, interval) [2], [25], [30]. Physical activity was evaluated subjectively through self-report or proxy activity questionnaires [23], [24], [26], [27], [29], [31]–[33] and/or objectively with motion and activity monitors (n = 5) [24], [26]–[29]. Studies described involvement in physical activities in terms of exertion level (light, moderate or vigorous), frequency (hr/day or hr/week) (n = 6) [24], [26]–[28], [31], [32], duration (months in a year) (n = 3) [24], [26], [27], energy expenditure (Metabolic equivalent of a task (MET)) (n = 2) [23], [33] and movement counts (n = 4) [24], [26]–[28]. Some authors also quantified physical activity using ordinal scales to indicate level of participation (how active) [29], [30].

There were 3 studies that focused on social activities [2], [25], [30], 10 that also reported on physical activities [23], [24], [26]–[33], and 5 that explored potential determinants of participation [2], [28], [29], [32], [33]. These are summarized below.

### Participation in social activities

Schanberg et al. conducted a longitudinal study assessing activity reduction among 41 youth with JIA (aged between 8 to 17 years) and found that 66% had restricted participation in social activities at least one day during the 2 month study [2]. Billings et al. showed that youth (aged ≥10 years) with severe disease reported fewer activities with family members in comparison to those with milder disease, as well as significantly fewer activities with friends compared to healthy controls [25]. Although, participants with mild/inactive disease reported fewer activities with friends compared to healthy controls, they took part in more family activities than healthy controls. Huygen et al.'s investigation into the psychological, behavioural and social adjustment of 47 children and adolescents with JIA revealed that there were no differences among adolescents (ages 12 to 16 years) in terms of the frequency of social interactions between those with JIA and healthy controls [30]. However, children (ages 7 to 11 years) with JIA did not play or visit as frequently with friends as their healthy peers (1.7 (0.1) versus 2.0 (0.0), p = 0.04; 3-point Likert scale) [30].

### Participation in physical activities

Results on physical activity measured through self-report in 7 studies were lower in children and adolescents with JIA compared to their healthy controls [23], [24], [26], [30]–[33]. Specifically participants with JIA participated less in moderate to vigorous physical activities compared to healthy peers [24], [32], however no differences were found between groups for participation in light physical activity [24], [26], [27], [32]. Studies examining participation in sports revealed that both children (range 4.6 to 11.0 years) and adolescents (range 11 to 18 years) with JIA took part in sports less frequently than their peers without JIA [24], [26], [30], [32]. Two studies reported fewer weight-bearing physical activities among those with JIA compared to healthy controls [23], [31]. However Félin et al. reported that only those with systemic JIA (and not those with polyarticular or oligoarticular JIA) were less involved in weight-bearing activities compared to healthy controls [23].

Significant within group differences for participation in physical activity were found in two studies [23], [27]. When exploring disease subtypes, authors reported that patients with systemic JIA (26.0 [3.8, 48.2] METs hours/week) were significantly less involved in weight-bearing PA compared to patients with oligoarticular (45.4 [20.9, 70.9] METs hours/week) or polyarticular (40.7 [23.8, 57.6] METs hours/week) JIA (*p*≤0.01) [23]. The other study described how duration of involvement in organized sports was significantly greater in children with normal bone mineral density (1.7 (2.2) months/year) in comparison to those with low bone mineral density (0.2 [0.8] months/year, p = 0.03) [27]. Only one study compared daily average physical activity between boys and girls in 30 patients with JIA and found no statistically significant differences [32].

When measured objectively through accelerometry, most authors reported no statistically significant differences in average daily physical activity (i.e. movement counts) between JIA and healthy groups [24], [26], [27]. In fact only Maggio et al. found that children and adolescents with JIA participated less in moderate to vigorous physical activity (MVPA) compared to healthy counterparts (JIA, 6.9% of time in MVPA, 54.1±5.7 min/day; healthy controls, 9.1% of time in MVPA, 71.3±4.5 min/day), p = 0.04 [28]. Furthermore, two studies indicated that fewer children with JIA met international recommendations of 60 minutes of daily MVPA compared to healthy controls [28], [32], and the proportion varied between one-third (23% vs. 66%) to two-thirds (38% vs. 60%) than that of healthy controls [28], [32].

### Potential determinants of leisure

Potential determinants of leisure participation were assessed in 5 studies [2], [28], [29], [32], [33], however only 4 reported statistically significant associations with leisure [2], [28], [29], [32] (Table 2). Socio-demographic factors were assessed in two studies [28], [29]. Results showed that when adjusted for age boys displayed higher physical activity levels than girls [28]. Also, the younger the child, the higher the level of physical activity [29]. Disease-related factors were assessed in all studies examining potential determinants [2], [28], [29], [32], [33]. However in only four studies were disease-related factors associated with lower levels of leisure activity (systemic JRA subtype, lower well-being, pain, larger number of painful joints, stiffness, and fatigue) [2], [23], [29]. Whereas longer disease duration was associated with more frequent participation in physical activity [32]. Anthropometric measures were also considered as potential determinants of physical activity participation in one study [28] and higher height and weight were associated with increased physical activity among children and adolescents living with JIA [28].

## Discussion

Our systematic review revealed that participation in social and physical activities during leisure-time may be decreased in children and adolescents living with JIA as compared to their healthy peers and fewer JIA patients met national physical activity recommendations. Studies to date have methodological weaknesses; therefore results should be interpreted with caution. Furthermore, only a few studies empirically explored the association between potential determinants and participation in leisure-time activities. Authors used various subjective and objective outcome measures with differing measurement units and scales and lack of validity and reliability to assess involvement in leisure-time activities making it difficult to generalise results on leisure participation in JIA.

The self-report assessment of leisure participation was for the most part restricted to a handful of activities, specifically social (e.g. taking part in play with friends, seeing friends, going to ball games, being part of a club, participating in sports, going to parties or dances) and physical (e.g. participating in organized sports or weight-bearing activities), rather than a larger gamut of recreational activities (e.g. playing board games or card games, screen time activities [computer or video games, watching TV], playing with pets), skill-based (e.g. swimming; learning to sing [choir or individual lessons], learning to dance, playing a musical instrument) and self-improvement (e.g. writing a story, reading, doing volunteer work, shopping). A more comprehensive approach to assessment is needed in order to truly capture a complete picture of leisure in this population. Findings would subsequently enable health care professionals to assess the benefits and potential determinants of participation. As our review demonstrates, social and physical activities of children and adolescents with JIA may vary with age, sex, type of activity, social engagement (friends, family) and disease activity/status.

In our review, social participation in young children was characterised as play with friends, whereas for adolescents it included analysis of spontaneous social contact and going on outings with friends. Among children, social interactions are often fostered through informal play time with friends [4], [34]. Children with JIA took part in less play than healthy peers possibly due to physical limitations and pain brought on by their disease. Younger children tend to be involved in more active types of play, which may discourage young patients with JIA affected by fluctuating joint pain and swelling to partake in these activities with friends [2], [29].

Adolescents with JIA took part in as many spontaneous social interactions as their healthy peers [30], however did not attend as many outings with friends [25]. These results parallel those found among adolescents (12 to 20 years of age) with cerebral palsy, where participants favoured quiet social activities such as hanging out, listening to music and talking on the phone with friends [35] and less in outings [35]. The similarities found between adolescents with and without JIA may reflect the natural progression of social leisure participation [4], [34]; as children get older they become less involved in play and more engaged in socially oriented activities which tend to be less physically straining [36], [37].

Social engagement in activities (i.e. with whom they engaged in activities) [38] varied across disease status [25]. The more severe the disease activity the less patients participated in activities with friends and family members [25]. During periods of more severe arthritis, parents may limit their child's participation in activities to avoid exacerbating disease symptoms. The fear of pain may influence the parents' and the child's willingness to take part in social activities [39], [40]. This avoidance and the need to monitor their child's health closely may also explain why those even with milder or inactive JIA may be less inclined to participate in social activities with friends and more involved in family activities [25]. Similarly, children and adolescents with systemic JIA (a more severe subtype of JIA) were less involved in weight-bearing physical activities compared to those with oligoarticular or polyarticular JIA [23].

The association of age and sex with participation in physical activity is often studied [41]. Our findings support that physical activity in JIA tends to decrease with age [29], which parallels results from the general pediatric population [4], [34]. Boys are often found to be more physically active than girls [37], [41]–[43]. Studies included in our review revealed divergent results. When adjusting for age (mean age 10.8±0.5 years; range 4.8 to 17.9) Maggio et al. (2010) reported that boys with JIA were more physically active than their female counterparts [28]. Whereas when comparing physical activity among adolescents with JIA (mean age of 17.0±0.6 years) Lelieveld et al. (2008) reported no significant differences across sexes [32]. In general, participation in sports and other active pursuits is higher throughout middle childhood and drops during adolescence [34]. This may in part explain why no significant differences in physical activity were found among adolescent boys and girls with JIA [32].

Although results varied, most studies showed that children with JIA spent significantly less time pursuing physical activity compared to healthy controls [23], [24], [26], [28], [30]–[33]. In addition to disease-related factors (e.g. disease duration, number of painful and swollen joints), poor physical fitness may also contribute to lower levels of physical activity in JIA [29]. Children living with polyarticular JIA have been found to be less physically fit than healthy and normally active (non-competitive) controls [44]. The lack of physical fitness is not necessarily associated with disease severity but avoidance of exercise by those with JIA is often encouraged by parents and health care professionals to limit exacerbation of disease related symptoms [44].

Osteopenia (or lower than normal bone mineral density) is of particular concern in children and adolescents with JIA as it may lead to decreased bone strength and a subsequent increased risk for fractures [45], [46]. Those suffering from JIA are at greater risk of bone density anomalies secondary to greater disease severity, treatment by glucocorticoids and decreased participation in organized sports or other physical activity [26], [27]. The more children took part in organized sports the better their bone mineral density [27]. The study's cross-sectional design restricts inference of causality and one can argue that low bone mineral density (a characteristic of more severe disease [26]) is a potential determinant of participation in sports rather than an outcome.

Self-report measures were a popular means of collecting data on social and physical leisure-time activities. Researchers may appreciate the low cost and convenience of using these tools. Activity monitors were also used albeit less frequently to objectively assess physical activity. Electronic data collection is not affected by recall bias and may be more appropriate for assessing physical activity among children and adolescents [47].

Less than 40% of those with JIA met international recommendations to engage in at least 60 minutes of moderate to vigorous physical activity daily, as compared to 60% of healthy controls [28]. Persons with JIA may intentionally avoid more physically strenuous activities to keep from aggravating disease-related symptoms such as swollen joints [29]. However, there is no evidence that activity in fact exacerbates symptoms [48] and current evidence supports that physical activity is beneficial in JIA in helping to reduce pain, the number of swollen joints, as well as improving overall aerobic endurance and bone health [11], [49]. Furthermore, there is a potential for exercise to favor immune function and help reduce chronic inflammation [50]–[53].

The literature on childhood disability supports that involvement in leisure activities may be influenced by intrinsic and extrinsic factors [3], [42]. Most studies in pediatric rheumatology explored the effects of disease severity as a function of disease-related factors (e.g. active joint count, pain, fatigue, function/disability), rather than potential effects of contextual factors relating to the child (personal) and his or her environment (family and community). Only one study explored the potential association of anxiety and depression with participation in social activities, however no significant results were found [33]. No studies reported on such aspects as self-esteem, motivation, activity preferences, family functioning and environmental barriers.

Future research determining extent of involvement in a range of leisure activities in children with JIA and their determinants may benefit from a well grounded theoretical framework such as the ICF, which considers how participation in activities is influenced by body structure and function associated with the health condition (e.g. active joint count, perceived pain), activity limitations as well as personal (e.g. age, sex, motivation) and environmental (e.g. family functioning, availability of community services) factors [8], [9].

### Limitations

Based on the appraisal criteria of the Quality Assessment Tool for Quantitative Studies [19], no study met all quality criteria. All studies retained for review were observational [20]. Most studies recruited children and adolescents with JIA systematically from a convenience sample (i.e. clinic), weakening external validity. Furthermore, sample sizes were all relatively small, which may lead to type 2 errors. We do recognise how challenging recruitment in pediatric rheumatology is, considering the low prevalence of the disease (range of 0.02 to 0.40 percent of children) [54]. Most studies used outcome measures with questionable psychometric properties threatening the quality of data collection [2], [24]–[27], [29], [31]–[33].

## Conclusions

In general, children and adolescents with JIA display limitations in participation in either social or physical leisure-time activities. Despite the known benefits of leisure participation, no study to date has fully explored the experience of leisure participation in JIA. In order to properly explore leisure participation in JIA, there is a need for high quality observational studies. Future research into the study of leisure participation in pediatric rheumatic diseases may benefit from using a comprehensive, valid and reliable outcome measure exploring a broad range of possible leisure activities from which to draw valid conclusions [55]. Moreover, the assessment of contextual factors may provide researchers with compelling information on potential barriers and facilitators to leisure in JIA.

## Supporting Information

Appendix S1 **Search strategy.** Appendix S1 presents the search strategy used to identify studies on leisure activities in JIA.
(DOCX)

Appendix S2 **Completed PRISMA checklist.** Appendix S2 presents the completed PRISMA checklist for the systematic review.
(DOCX)
