# Supplementary material for: Leisure in Children and Adolescents with Juvenile Idiopathic Arthritis: A Systematic Review
Source: PLoS One. 2014 Oct 20;9(10):e104642. doi: 10.1371/journal.pone.0104642 (PMC4203655; doi:10.1371/journal.pone.0104642)
Supplement: Appendix S1 — Search strategy. Appendix S1 presents the search strategy used to identify studies on leisure activities in JIA. (DOCX) [file pone.0104642.s001.docx]

# Appendix S1

**Search strategy**

A search strategy will be developed in consultation with a librarian (GB) and will be applied to the following electronic databases: Medline (1946 to week May week 3 2013)^[[1]](#footnote-1)^, the Cochrane library^[[2]](#footnote-2)^, EMBASE (1974 to 2013 Week 21), Cumulative Index of Nursing and Allied Health Literature (CINAHL) (1982 to December week 1 2013), Base de Données en Santé Publique (June 2013), [Education Resources Information Center](http://eric.ed.gov/) (ERIC) (1965 to April 2013), [Health and Psychosocial Instruments](http://ovidsp.tx.ovid.com/sp-3.8.1a/ovidweb.cgi?&S=EIGJFPNOPGDDKFENNCOKDBGCFGJFAA00&New+Database=Single%7c25) (1985 to April 2013), OT Seeker and [PsycINFO](http://ovidsp.tx.ovid.com/sp-3.8.1a/ovidweb.cgi?&S=EIGJFPNOPGDDKFENNCOKDBGCFGJFAA00&New+Database=Single%7c33) (1806 to May Week 3 2013).

The following search strategy has been formulated with the help of a librarian. It will be used for OVID MEDLINE but will be modified for each database:

Database: Ovid MEDLINE(R) In-Process & Other Non-Indexed Citations and Ovid MEDLINE(R) <1946 to Present>

Search Strategy:

--------------------------------------------------------------------------------

1 juvenile idiopathic arthritis.mp. or exp Arthritis, Juvenile Rheumatoid/ (8644)

2 juvenile arthritis.mp. (618)

3 juvenile chronic arthritis.mp. (1028)

4 juvenile ankylosing spondylitis.mp. (82)

5 juvenile oligoarthritis.mp. (12)

6 juvenile polyarthritis.mp. (47)

7 juvenile psoriatic arthritis.mp. (56)

8 juvenile enthesitis related arthritis.mp. (1)

9 juvenile systemic arthritis.mp. (0)

10 1 or 2 or 3 or 4 or 5 or 6 or 7 or 8 or 9 (9027)

11 physical activity.mp. or exp Motor Activity/ (199316)

12 physical activities.mp. (3356)

13 motor activities.mp. (841)

14 leisure.mp. or exp Leisure Activities/ (144832)

15 leisure activity.mp. or exp Leisure Activities/ (139612)

16 leisure participation.mp. (52)

17 leisure involvement.mp. (8)

18 social integration.mp. (1547)

19 participation.mp. or exp Social Participation/ (112737)

20 social activity.mp. (1040)

21 social activities.mp. (2111)

22 exp "Play and Playthings"/ or play*.mp. (725984)

23 sport*.mp. or Racquet Sports/ or Sports/ or Snow Sports/ (59674)

24 exercis*.mp. (238137)

25 exp Recreation/ or recreation*.mp. (129542)

26 accelerometer*.mp. or exp Actigraphy/ (5328)

27 accelerometry.mp. or exp Accelerometry/ (2390)

28 exp Walking/ or Walk*.mp. (71277)

29 exp Monitoring, Ambulatory/ or activity monitor*.mp. (22144)

30 pedometer*.mp. (1266)

31 actigraph*.mp. (2906)

32 sports participation.mp. (877)

33 play participation.mp. (2)

34 exp Swimming/ or aquatic sports.mp. (17662)

35 winter sports.mp. (173)

36 exp Bicycling/ or bicycle*.mp. (15539)

37 biking.mp. (272)

38 cycling.mp. (33268)

39 11 or 12 or 13 or 14 or 15 or 16 or 17 or 18 or 19 or 20 or 21 or 22 or 23 or 24 or 25 or 26 or 27 or 28 or 29 or 30 or 31 or 32 or 33 or 34 or 35 or 36 or 37 or 38 (1336273)

40 10 and 39 (518)

1. [Ovid MEDLINE(R) In-Process & Other Non-Indexed Citations and Ovid MEDLINE(R)](http://ovidsp.tx.ovid.com/sp-3.8.1a/ovidweb.cgi?&S=EIGJFPNOPGDDKFENNCOKDBGCFGJFAA00&New+Database=Single%7c29) 1946 to Week 3 May 2013 [↑](#footnote-ref-1)
2. EBM Reviews - Cochrane Database of Systematic Reviews (2005 to March 2013), EBM Reviews - ACP Journal Club (1991 to April 2013), EBM Reviews - Database of Abstracts of Reviews of Effects (2nd Quarter 2013), EBM Reviews - Cochrane Central Register of Controlled Trials (March 2013), EBM Reviews - Cochrane Methodology Register (3rd Quarter 2012), EBM Reviews - Health Technology Assessment (2nd Quarter 2013), EBM Reviews - NHS Economic Evaluation Database (2nd Quarter 2013) [↑](#footnote-ref-2)
